# CD200R1 and CD200R1L expression is regulated during B cell development in swine and modulates the Ig production in response to the TLR7 ligand imiquimoid

**DOI:** 10.1371/journal.pone.0251187

**Published:** 2021-05-07

**Authors:** Teresa Poderoso, Paloma Martínez De la Riva, Belén Álvarez, Ángel Ezquerra, Javier Domínguez, Concepción Revilla

**Affiliations:** Departamento de Biotecnología, Instituto Nacional de Investigación y Tecnología Agraria y Alimentaria (INIA), Madrid, Spain; Emory University School of Medicine, UNITED STATES

## Abstract

The CD200R family comprises a group of paired receptors that can modulate the activation of immune cells. They are expressed both on myeloid cells and lymphocyte subsets. Here we report that the expression of these receptors on porcine B cells is tightly regulated, being mainly expressed on mature cells. The expression of the inhibitory receptors CD200R1 and/or its splicing variant CD200R1X2, either in combination or not with the activating receptor CD200R1L, is upregulated in sIgM^+^ effector/memory cells, and tends to decline thereafter as these cells progress to plasmablasts or switch the Ig isotype. sIgM^+^ naïve and primed cells only express, by contrast, the CD200R1X2 receptor. B-1 like cells also express CD200R1 isoforms, either alone or in combination with CD200R1L. Treatment of peripheral blood mononuclear cells with a monoclonal antibody specific for inhibitory receptors, enhances the IgM and IgG production induced by TLR7 stimulation suggesting a modulatory role of B cell functions of these receptors.

## 1. Introduction

Cells of the immune system express a variety of paired receptors characterised by sharing a high sequence identity in their extracellular regions while differing in their transmembrane and cytosolic domains, which trigger opposing inhibitory and activating pathways [1]. Signals from these receptors can synergize with, antagonize or modulate those from other receptors, and their opposing functions provide the immune system with a mechanism to “fine-tune” innate and adaptive immunity, ensuring an effective response against pathogens while restraining detrimental effects to the host.

CD200R1 and CD200R1L belong to the CD200R family of paired receptors. Both are transmembrane type I glycoproteins, with two Ig superfamily domains in their extracellular regions [2]. CD200R1 contains three tyrosine residues in its cytoplasmic tail, through which it can recruit the adaptor molecule Dok2 and activate RasGAP, leading to inhibition of the ERK signaling pathway [3–5]. On the other hand, CD200R1L contains a lysine residue in the transmembrane segment, through which it associates with DAP12, a signaling protein with an immunoreceptor tyrosine-based activation motif in its cytoplasmic tail that recruits and activates tyrosine kinases leading to cell activation [2, 6].

CD200R1 and its ligand CD200 interact through their respective N-terminal Ig domains [7]. However, the binding of CD200R1L to CD200 remains controversial [8, 9].

In humans and mice, CD200R1 and CD200R1L are predominantly expressed on myeloid cells (monocytes, granulocytes, macrophages and dendritic cells) but also on NK cells and of T and B lymphocyte subsets [2, 10, 11]. Whereas several studies have addressed the role of these molecules in myeloid cells, information on their function in B cells is rather scarce. In humans, CD200R1 has been shown to be mainly expressed on terminally differentiated B cells, which has led to suggest that it might be involved in avoiding reactivation of memory or effector B cells, rather than in setting a threshold for activation of naïve B cells [11].

Progress in the characterisation of developmental pathways of B cells in swine has been hindered by the shortage of specific reagents. Moreover, markers used to identify memory B cells in other species, like CD27 in humans, [12, 13] are not expressed on porcine B cells [14]. Sinkora and colleagues classified porcine B cell into four subsets on the basis of CD2 and CD21^b^ expression, being CD21^b^ recognized by an anti-bovine anti-CD21 mAb on porcine cell: naïve (CD2^+^CD21^b+^), primed (CD2^-^CD21^b+^), effector/memory (CD2^+^CD21^b-^) and plasmablasts (CD2^-^CD21^b-^) [15, 16]. Moreover, two putative porcine B1-like subsets have been defined as CD21^-^IgM^high^CD11R1^+^ and CD21^-^IgM^high^CD11R1^-^ B cells, showing CD11R1^+^ B-1 like cells the highest proliferative capacity in response to TLR7 signaling [17], which has been shown to be regulated by CD200R1 [18].

We have recently characterised two monoclonal antibodies (mAbs), named PCT1 and PCT3. PCT1 reacts with both CD200R1 and CD200R1L, while PCT3 recognizes CD200R1 and a splicing variant of this receptor, named CD200R1X2, which lacks the V-type Ig-like domain. Used in combination, these mAbs allow to study CD200R1 and CD200R1L expression in swine, showing that these receptors are expressed on a fraction of peripheral blood B cells [19].

The aim of the present study has been to characterise the distribution of CD200R1 and CD200R1L receptors on porcine B cell subsets and to analyse the effect of their engagement with mAbs on Ig production triggered by TLR7 stimulation.

## 2. Materials and methods

### 2.1. Animals and cells

Blood samples were obtained from 6 to 12-month-old female outbred Large-White pigs, acquired from a commercial high health status herd, and maintained in a conventional farm facility at the Departamento de Reproducción Animal, INIA, Madrid. For bleeding (50–100 ml), pigs were restrained, and the procedure was carried out without anaesthesia and aseptically, by punction in the cranial vena cava. Peripheral blood mononuclear cells (PBMC) were isolated on Percoll discontinuous gradients, after blood sedimentation in dextran. Erythrocytes were lysed by hypotonic treatment. Finally, cells were resuspended in complete medium (RPMI 1640 medium containing 10% Foetal bovine serum, 2mM L-glutamine, 5 x 10^−5^ 2-mercaptoethanol and 50 μg/ml gentamicin). All experiments were performed with fresh cells.

All experiment protocols were approved by the Ethical and Animal Welfare committees of the Instituto Nacional de Investigación y Tecnología Agraria y Alimentaria (Resolution number: CEEA 2014/035b), and the Dirección General de Agricultura, Ganadería y Alimentación de la Comunidad de Madrid (Resolution number: Proex 040/15). No anaesthesia, euthanasia, or any kind of animal sacrifice has been used in the study.

### 2.2. Flow cytometric analysis

In this study, two different anti-CD21 mAbs were used, BB6-11C9.6 and IAH-CC51. BB6-11C9.6 mAb is a pan-CD21 mAb while IAH-CC51, an anti-bovine CD21 mAb cross-reactive with porcine CD21 (CD21^b+^), stains only a fraction of porcine CD21^+^ B cells which have been related to naïve and primed B cells [16]. Anti-leukocyte receptor mAbs used in this study are summarized in Table 1.

**Table 1:** Characteristics of mAbs used in this study.

Clone	Host and Specificity	Isotype	Source
MSA4	mouse anti-porcine CD2	IgG2a	Kingfisher Biotech
BB23-8E6	mouse anti-porcine CD3	IgG2b	M. Pescovitz, Indiana Univ. USA
MIL-4	mouse anti-porcine CD11R1	IgG1	BIO-RAD
BB6-11 C9.6	mouse anti-porcine CD21	IgG1	Kingfisher Scientific
1AH-CC51	mouse anti-bovine CD21 (CD21^b^)	IgG2b	Kingfisher Scientific
74-22-15a	mouse anti-porcine CD172a	IgG2b	J. Lunney, ARS, USDA
5C9	mouse anti-porcine IgM	IgG1	ATCC
K139.3E1	mouse anti-porcine λ light chain	IgG2a	K. Haverson, Univ. Bristol, UK
PCT1	mouse anti-porcine CD200R1 and CD200R1L	IgG1	Own production, INIA
PCT3	mouse anti-porcine CD200R1 and CD200R1X2	IgG2a	Own production, INIA

For analysis of the distribution of CD200R1 isoforms and CD200R1L in B cell subsets defined on the basis of CD2 and CD21^b^ expression, PBMCs (1 x 10^6^/well) were incubated with PCT1, anti-CD2 and anti-CD21^b^ mAbs. After two washes in PBS containing 0.1% BSA and 0.01% sodium azide (FACS buffer), APC-Cy7-conjugated goat anti-mouse IgG1, PE-Cy7-conjugated goat anti-mouse IgG2a and APC-conjugated goat anti-mouse IgG2b polyclonal antibodies (all from Southern Biotech, USA) were added. Afterwards, cells were washed and free binding sites blocked with 10% normal mouse serum. Finally, they were incubated with Alexa 488-conjugated anti-IgM and biotinylated PCT3 mAbs, followed by streptavidin-PE (BD Biosciences, USA).

To examine the reactivity of PCT1 and PCT3 mAbs on B cell subpopulations defined according to the expression of CD21 and CD11R1, PBMCs (1 x 10^6^/well) were incubated with anti- porcine CD21 and PCT3 mAbs followed by staining with PE-conjugated goat anti-mouse IgG1 and PE-Cy7-conjugated goat anti-mouse IgG2a polyclonal antibodies (both from Southern Biotech, USA). After blocking free binding sites of secondary antibodies with normal mouse serum, cells were incubated with Alexa 488-conjugated anti-IgM, Alexa 633-conjugated anti-CD11R1 and biotinylated PCT1 mAbs, followed by streptavidin-BV421 (BD Biosciences, USA).

For analysis of the reactivity of PCT1 and PCT3 mAbs in B cell subsets defined by the expression of sIgM and λ light chain (Lλ), PBMCs (1 x 10^6^/well) were incubated with PCT1 and anti-λ chain mAbs. After washing with FACS buffer, APC-Cy7-conjugated goat anti-mouse IgG1 and APC-conjugated goat anti-mouse IgG2a polyclonal antibodies (both from Southern Biotech, USA) were added. Next, free binding sites were blocked with 10% normal mouse serum and cells incubated with Alexa 488-conjugated anti-IgM and biotinylated PCT3 mAbs, followed by streptavidin-PE (BD Biosciences, USA).

Doublets were excluded by gating using FSC-A vs FSC-H plots and dead cells by staining with Sytox blue (Life Technologies, USA). Irrelevant isotype-matched mAbs, unlabelled or labelled with biotin, Alexa-488 or Alexa-633, were used as negative controls.

Cells were analysed in a FACSCanto II flow cytometer (Becton Dickinson, USA). At least 100,000–150,000 lymphocytes were acquired. Data were analysed with FlowJo software FlowJo_v10.6.1.

### 2.3. In vitro Ig production

PBMC were seeded in round-bottom 96-well plates at 4 × 10^5^ cells/well in a final volume of 200 μl of complete medium alone or containing 2 μg of PCT1 or PCT3 mAbs or isotype-matched control mAbs. Cells were stimulated with 5μg/ml Imiquimoid (Invitrogen, USA) or left unstimulated. Cultures were carried out at 37°C and in a 5% CO_2_ atmosphere. After a week, supernatants were collected, and stored frozen at −80°C until assessment of Ig production. Supernatants were analysed for IgM and IgG production by using a commercial ELISA kit from Bethyl (USA) following the manufacturer’s instructions.

### 2.4. Statistical analysis

Statistical analyses were performed with Graphpad Prism 5 software (Graphpad, San Diego, CA). One way ANOVA followed by Bonferroni adjustment was used to assess statistically significant differences between groups. Symbols used: *, p≤0.05; **, p≤0.01, ***, p≤0.001.

## 3. Results

### 3.1 Analysis of the expression of CD200R1 isoforms and CD200R1L in peripheral blood B cell subsets

Previous studies in our laboratory have shown that porcine CD200R1 isoforms and/or CD200R1L are expressed on a significant proportion of peripheral blood B cells [19]. A summary of the expression of porcine CD200R1, CD200R1X2 and CD200R1L receptors according to the labelling with PCT1 and PCT3 mAbs is depicted in Table 2.

**Table 2:** Summary of the expression of porcine CD200R1, CD200R1L and CD200R1X2 receptors defined by different patterns of PCT1 and PCT3 mAb staining [19].

Phenotype	Pattern of expression of CD200R family receptors
PCT1^-^PCT3^+^	CD200R1X2^+^
PCT1^+^PCT3^-^	CD200R1L^+^
PCT1^+^PCT3^+^	CD200R1 either alone or with any combination of CD200R1X2 and/or CD200R1L; or CD200R1L plus CD200R1X2
PCT1^-^PCT3^-^	CD200R1^-^CD200R1L^-^CD200R1X2^-^

**Fig 1:**
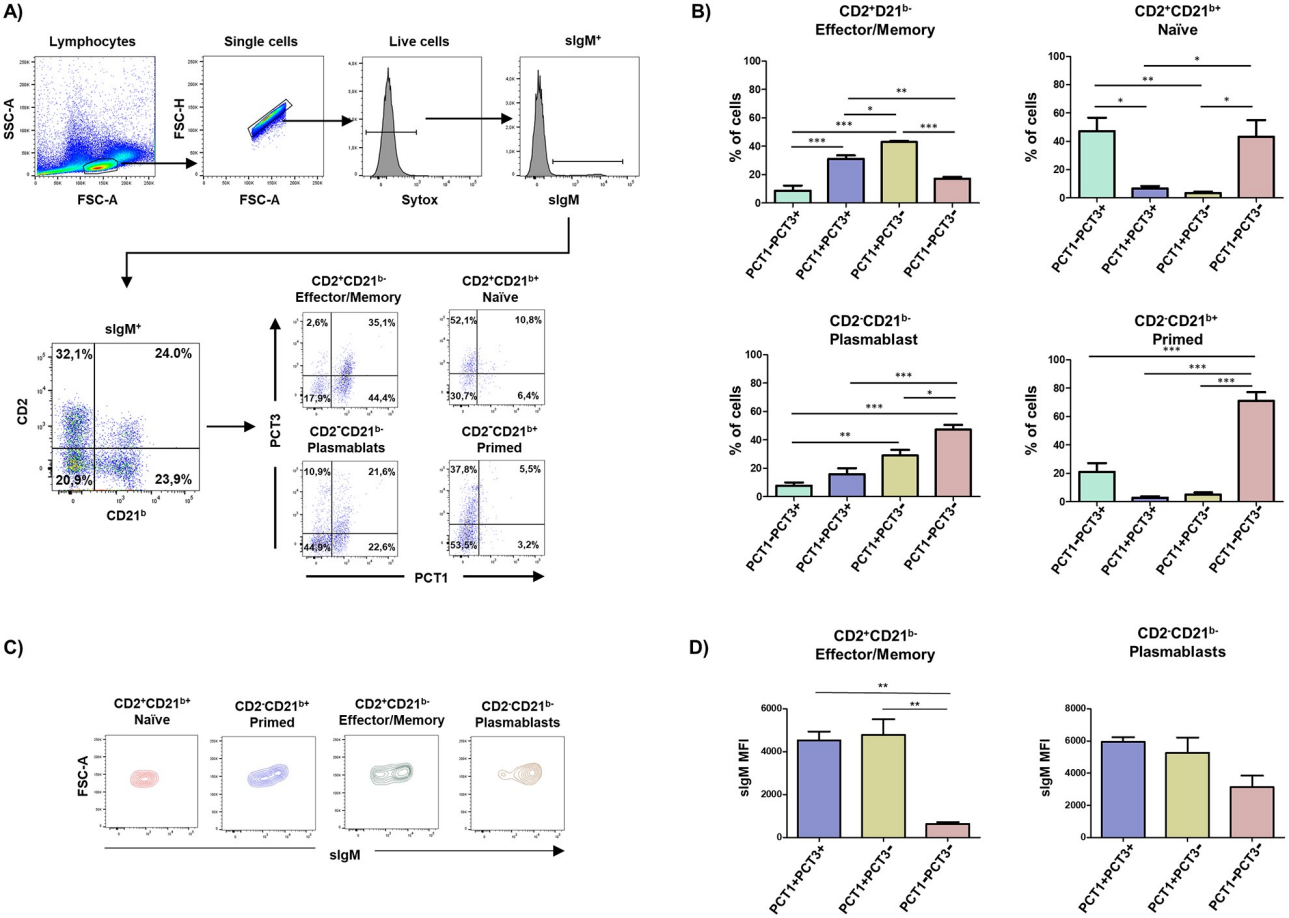
PCT1 and PCT3 mAb reactivity on sIgM^+^ B cell subsets. A) Blood lymphocytes were gated according to their FSC-A/SSC-A profiles. After excluding doublets and dead cells, sIgM^+^ cells were gated and analysed for the expression of CD2 vs CD21^b^. CD2/CD21^b^ defined cell subsets were further subgated for analysis of PCT1 vs PCT3 expression. Numbers indicate the percentage of cells within the respective quadrants. B) Graphs show average ± SEM of the percentage of cells in each cell subset analysed in 4 independent experiments carried out with cells from different animals. C) Contour-plots represent FSC-A vs. sIgM in the indicated B cell subsets. D) Graphs show average ± SEM of the sIgM mean fluorescence intensity of 4 independent experiments. One way ANOVA followed by Bonferroni adjustment was used to assess statistically significant differences. Symbols used: *, p≤0.05; **, p≤0.01, ***, p≤0.001.

To determine which subpopulations of B cells express CD200R1 isoforms and CD200R1L, we studied porcine peripheral blood sIgM^+^ B cell subsets proposed by Sinkora and Butler on the basis of CD2 and CD21^b^ expression [15, 16]. When we analysed the reactivity of PCT1 and PCT3 mAbs on these subpopulations (Fig 1A and 1B), naïve (CD2^+^CD21^b+^) and primed (CD2^-^CD21^b+^) B cells were mostly PCT1 negative, and therefore they do express neither CD200R1 nor CD200R1L at the cell surface. However, a substantial proportion of these cells (≈ 45% of naïve and 20% primed) were stained by PCT3 mAb, and so they presumably express the CD200R1X2 isoform.

Most effector/memory (CD2^+^CD21^b-^) cells were recognized by mAb PCT1, and about 40% of them were PCT3 negative (Fig 1A and 1B). These PCT1^+^PCT3^-^ cells most likely only express the CD200R1L receptor. Around 30% of effector/memory cells were PCT1^+^PCT3^+^, and might express CD200R1 either alone or in combination with CD200R1X2 and/or CD200R1L; alternatively, they might express CD200R1L plus CD200R1X2. There was also a minor fraction (≈20%) of these effector B cells which were negative for both PCT1 and PCT3.

With regard to plasmablasts (CD2^-^CD21^b-^), around half of them are PCT1^-^PCT3^-^ being negative for both CD200R1 and CD200R1L (Fig 1A and 1B). Nevertheless, about 25% are PCT1^+^PCT3^-^, expressing only the activating molecule CD200R1L, while other 20% are stained by both mAbs.

As previously described [16], CD21^b-^ B cells showed a larger size than CD21^b+^ cells (Fig 1C). Likewise, CD21^b-^ B cells expressed higher levels of sIgM than CD21^b+^ cells (Fig 1C). Among CD21^b-^ B cells, the highest levels of sIgM were found on the PCT1^+^ cells (Fig 1D).

**Fig 2:**
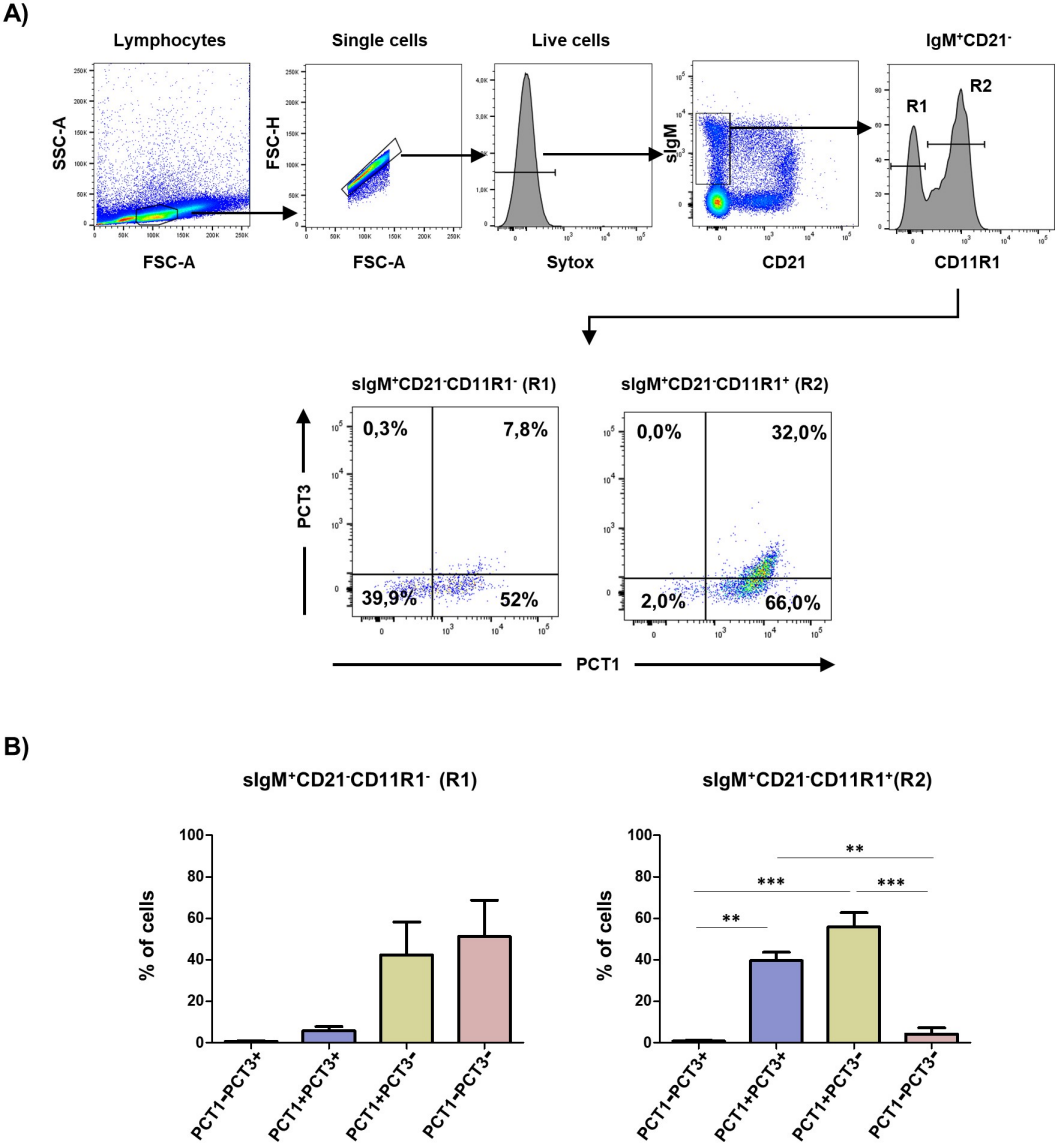
A) Blood lymphocytes were gated by FSC-A/SSC-A parameters and single and live cells selected. Then, binding of PCT1 and PCT3 mAbs was analysed on sIgM^+^CD21^-^CD11R1^+^ and sIgM^+^CD21^-^CD11R1^-^ B cell subsets. The anti-porcine CD21 mAb (clone BB6-11C9.6) was used. Numbers indicate the percentage of cells within the respective quadrants. B) Graphs show average ± SEM of the percentage of cells in each cell subset analysed in 3 independent experiments carried out with cells from different animals. One way ANOVA followed by Bonferroni adjustment was used to assess statistically significant differences. Symbols used: **, p≤0.01, ***, p≤0.001.

Porcine sIgM^+^ B cells can also be divided into different subsets according to the expression of CD21, recognized by the pan-CD21 mAb (clone BB6-1C9.6), and CD11R1. sIgM^+^CD21^-^CD11R1^+^ and sIgM^+^CD21^-^CD11R1^-^ cells have been proposed to comprise B1-like cells. Both sIgM^+^CD21^-^ cells produce high levels of IgM when stimulated with the TLR7 ligand resiquimoid but only CD11R1^+^ cells display a high proliferative response [17]. Most of these CD11R1^+^cells were positive for PCT1, being a substantial proportion of them (≈40%), also recognized by PCT3 (Fig 2B). These PCT1^+^PCT3^+^ might therefore express CD200R1 either alone or together with CD200R1X2 and/or with the activating receptor CD200R1L, or a combination of CD200R1L and the isoform of inhibitory receptor CD200R1X2. On the other hand, the other B1-like cell population, sIgM^+^CD21^-^CD11R1^-^ cells, was mostly PCT3^-^, being about half of them also negative for PCT1 (Fig 2B). sIgM^+^CD21^-^CD11R1^-^PCT1^-^PCT3^-^ cells would express neither CD200R1 nor CD200R1L isoforms, and the PCT1^+^PCT3^-^ subset would only express CD200R1L.

**Fig 3:**
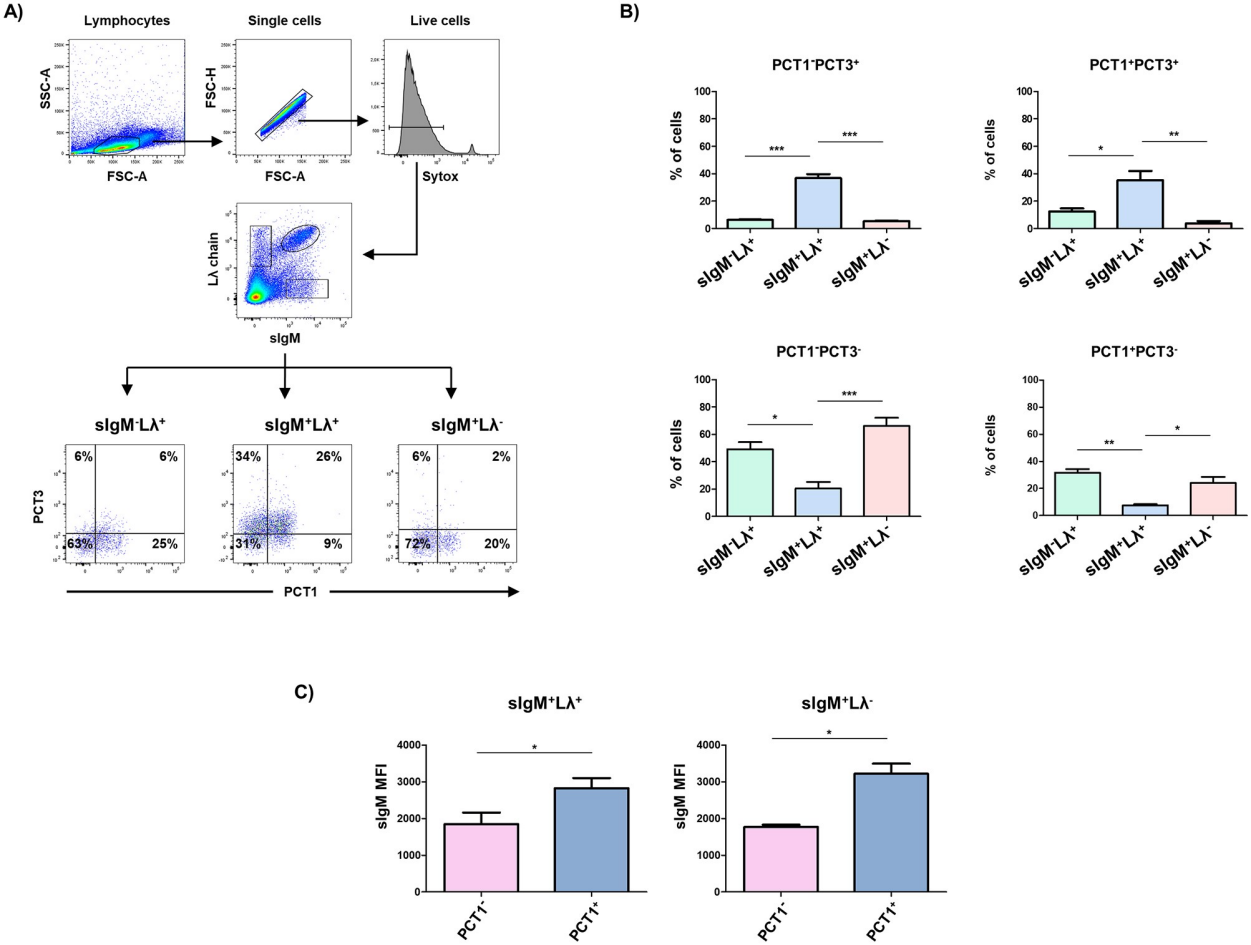
A) Blood lymphocytes were gated by FSC-A/SSC-A parameters and single and live cells selected. Then, binding of PCT1 and PCT3 mAbs was analysed on sIgM^-^Lλ^+^, sIgM^+^Lλ^+^ and sIgM^+^Lλ^-^ B cell subsets. Numbers indicate the percentage of cells within the respective quadrants. B) Graphs show average ± SEM of the percentage of cells in each cell subset analysed in 4 independent experiments carried out with cells from different animals. C) Graphs show average ± SEM of the sIgM mean fluorescence intensity of 4 independent experiments. One way ANOVA followed by Bonferroni adjustment was used to assess statistically significant differences. Symbols used: *, p≤0.05; **, p≤0.01, ***, p≤0.001.

We also examined the expression of CD200R1 isoforms and CD200R1L in B cell subsets defined by the expression of sIgM vs λ light chain (Lλ) (Fig 3A and 3B). sIgM^-^Lλ^+^ B cells, which correspond to class-switched cells, were mostly PCT3 negative, with a minor fraction of them positive for PCT1, which would only express CD200R1L. Within the sIgM^+^Lλ^+^ subset, a high proportion (60%) were positive for PCT3, being approximately half of them also positive for PCT1. These double positive cells might express CD200R1 either alone or in combination with CD200R1X2 and/or CD200R1L; alternatively, they might express CD200R1X2 together with CD200R1L. The other half, negative for PCT1, would express only the CD200R1X2 isoform of the inhibitory receptor. Interestingly, sIgM^+^Lλ^-^ B cells display a pattern of expression of these receptors similar to that of class-switched sIgM^-^λ^+^ B cells. The analysis of sIgM levels in PCT1^+^ vs PCT1^-^ cells showed that sIgM expression was also higher in PCT1^+^ than in PCT1^-^ cells in both sIgM^+^Lλ^+^ and sIgM^+^Lλ^-^ B cell subsets (Fig 3C).

### 3.2 Effect of CD200R1 and/or CD200R1L receptor engagement on Ig production induced by TLR7 stimulation

**Fig 4:**
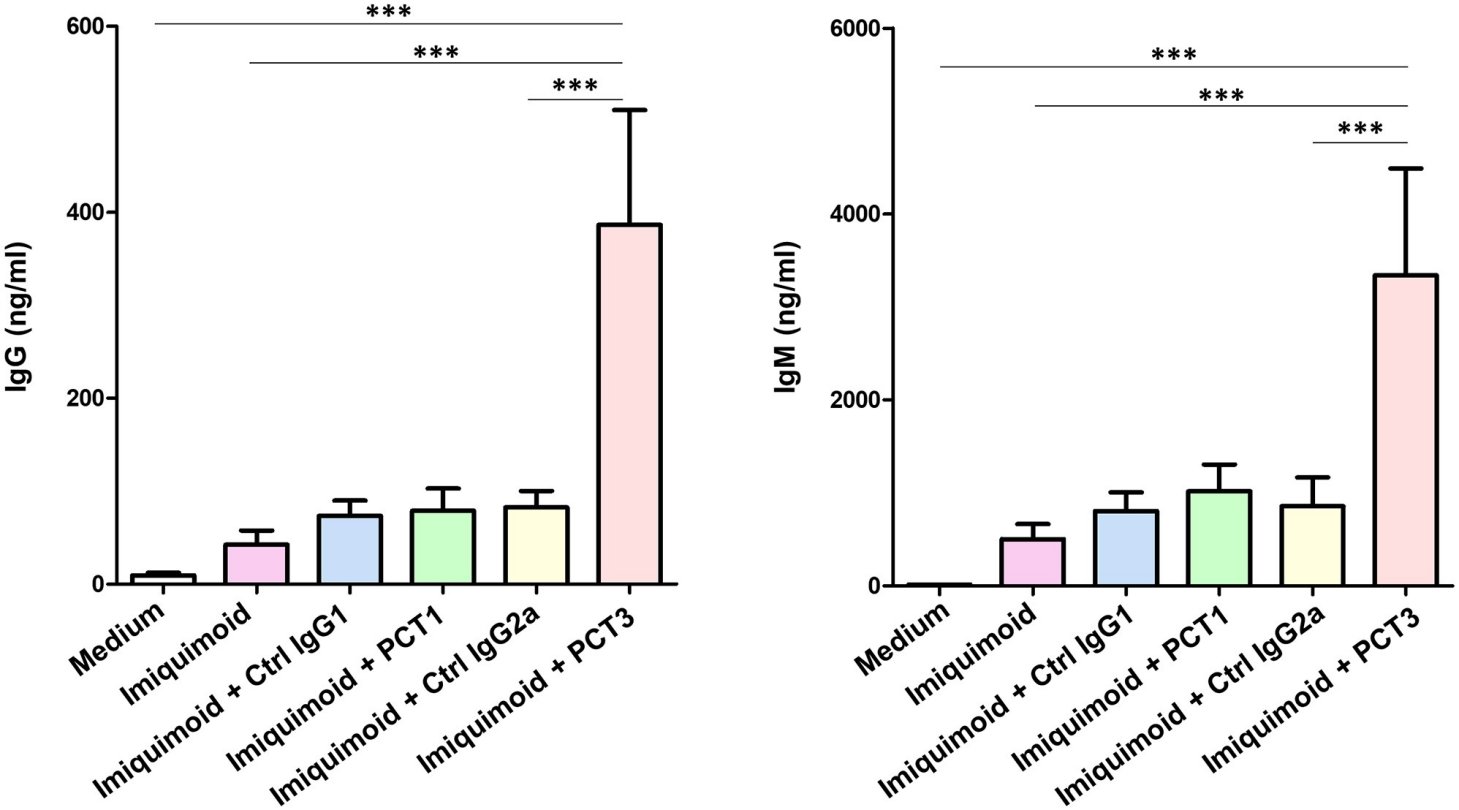
IgG and IgM production after stimulation of PBMC with the TLR7 ligand imiquimoid in the presence of PCT1, PCT3 or isotype-matched control mAbs or medium alone. After seven days supernatants were collected and analysed by ELISA for antibody production. Results shown mean ± SEM from 6 independent experiments. One way ANOVA followed by Bonferroni adjustment was used to assess statistically significant differences. Symbols used: ***, p≤0.001.

CD200R1 has been shown to inhibit, in human HEK293 cells, the NF-kB activity induced by TLR7 stimulaton with imiquimoid [18]. Porcine peripheral blood B cells express high levels of TLR7 and its triggering induce activation, proliferation, and differentiation of B cells into antibody secreting cells, [17]. So, we next examined the effect of CD200R1/CD200R1L engagement, with PCT1 or PCT3 mAbs, on the antibody production, after stimulation with the TLR7 ligand imiquimod. PBMC were stimulated with 5μg/ml of imiquimoid in the presence of PCT1, PCT3 or isotype-matched control mAbs or left unstimulated. After seven days of culture, supernatants were collected and analysed for the presence of IgM and IgG. Treatment with mAb PCT3, but not mAb PCT1, induced an increase in the level of IgM and IgG secreted in response to imiquimod, with values 4 to 5-fold over those of control treated with the isotype control mAbs (Fig 4).

## 4. Discussion

Here we show that CD200R1 and CD200R1L, identified through the combined use of mAbs PCT1 and PCT3, are mainly expressed on porcine effector/memory B cells and plasmablasts. Most naive and primed B cells are negative for these receptors or express only the CD200R1X2 isoform (≈50% of naïve cells and ≈20% of primed B cells). The expression of inhibitory receptors (CD200R1 and/or CD200R1X2), either alone or in combination with activating CD200R1L form is higher in effector/memory cells compared to primed cells, and then it tends to decline as these cells switch the Ig isotype or progress towards plasmablasts. This downregulation of inhibitory receptors precedes that of the activating receptor, CD200R1L, which is still detectable in a significant proportion of these cells. Like sIgM^+^ plasmablasts, approximately 25% of the class-switched sIgM^-^Lλ^+^ B cells express only the activating receptor, CD200R1L. Nevertheless, the majority of class-switched B cells and plasmablasts do not express either CD200R1 isoforms or CD200R1L.

PCT1 and PCT3 staining reveals the existence of further heterogeneity within the populations of porcine sIgM+ effector/memory B cells and plasmablasts, allowing to discriminate three main subsets in each of them whose developmental relationship remains to be investigated. These subsets may also differ in the threshold of activation which may be regulated by the level of expression of CD200R1 and CD200R1L. Besides, the level of sIgM is higher in the PCT1^+^ than in the PCT1^-^ effector/memory cells, which may be related to the subsets identified in human within the sIgM^+^IgD^+^CD27^+^ memory B cells according to the levels of sIgM expression [20, 21]: the sIgM^hi^ B cells having similarities with marginal zone B cells that passed through germinal centers, and the sIgM^lo^ B cells being a less differentiated subset.

In adult animals (>6 months) all IgM^+^ B cells have been reported to be positive for CD21, recognized by BB6-11C9.6 mAb [16]. However, in our study and in agreement with data from Braun et al., 2017 [17], using the same anti-CD21 mAb, two populations of CD21^-^ cells, which represent B1-like cells, were detected: sIgM^+^CD21^-^CD11R1^+^ and sIgM^+^CD21^-^CD11R1^-^ cells. Compared to the IgM^+^CD21^-^CD11R1^+^ population that is composed of PCT1^+^PCT3^+^ and PCT1^+^PCT3^-^ cells, the IgM^+^CD21^-^CD11R1^-^ subset mostly contains PCT3^-^ cells, that therefore do not express inhibitory receptors, about half of which express CD200R1L (PCT1^+^PCT3^-^ cells).

A striking difference was observed in the expression of CD200R1 and CD200R1L receptors when sIgM^+^ B cells were split according to the expression of Lλ chain. Whereas, a high proportion of the sIgM^+^Lλ^+^ cells are PCT3^+^, which means that they express inhibitory receptors, most sIgM^+^Lλ^-^ cells, on the contrary, are PCT3^-^, displaying a pattern of reactivity with these mAbs similar to that of class-switched B cells (sIgM^-^Lλ^+^). About one third of sIgM^+^Lλ^-^ cells are PCT1^+^ and therefore they express only the activating receptor CD200R1L. This finding may reflect the existence of different control mechanisms that regulate the differentiation of porcine lambda and kappa B cells. In this regard, studies on porcine B cell development in bone marrow have shown that many IgLκ^+^ B cell precursors arise later than IgLλ^+^ B cell precursors and pointed to differences in developmental checkpoints between them [22–24].

In humans and mice, B cells express a variety of cell surface inhibitory receptors (PD-1, LAG-3, CD22, LAIR-1, FCRH4, CD300a, etc), whose expression differs depending on their developmental stage or activation status [25–30]. Thus, PD-1 regulates germinal center B cell development, limiting the amount of T cell help and shaping the quality and quantity of memory B cell population [31, 32]. Likewise, CD300a is predominantly expressed on a subset of memory B cells and plasmablasts/plasma cells and is capable of down-modulating B cell receptor-mediated signals [29]. A variety of studies have strongly suggested that CD200R1 acts as an inhibitory receptor that negatively regulates cell functions, particularly in cells of the myeloid lineage [18, 33]. Binding of CD200R1 to its ligand CD200, which is constitutively expressed on a broad variety of hematopoietic and non-hematopoietic cells, results in delivery of an inhibitory intracellular signal to the cell that bears CD200R1. One of the activation pathways that is regulated by CD200R1 in these cells is that triggered by TLR7 [18]. Since porcine B cells constitutively express TLR7, and its stimulation leads to their proliferation and differentiation into Ab-secreting cells [17], we tested the effect of ligation of CD200R1 and CD200R1L receptors with mAbs on “in vitro” Ig production. In apparent contrast with a role of CD200R1 as inhibitory receptor, treatment of PBMC with PCT3 results in an enhancement of the secretion of IgM and IgG induced by TLR7 stimulation. Treatment with mAb PCT1 does not have any effect on the Ig production. It is possible that B cells might be constitutively inhibited by constant interaction of CD200R1 with CD200, and that PCT3 mAb blocks this interaction. PCT3 appears to bind a sequence at the N-terminus of CD200R1 prior to the Ig-V domain, whereas PCT1 recognizes an epitope in the N-terminal V-type Ig domain [19]. Besides, PCT3 only reacts with CD200R1 isoforms, whereas PCT1 binds to both CD200R1 and CD200R1L receptors, and its effect may depend on the balance of signals delivered through these receptors.

The functional significance of the CD200R1X2 isoform expressed on a fraction of naïve and primed B cells is at present unknown. CD200R1X2 lacks the Ig-V domain [19], which has been shown to be critical for binding to CD200 [8], but keeps the Ig-C2 domain and the cytosolic tail involved in signaling transduction, so possible interactions with not yet characterised ligand/s cannot be ruled out.

In summary, the expression of CD200R1 and CD200R1L varies among different porcine peripheral blood B cell subsets, being predominantly found on effector/memory B cells and plasmablasts. This restricted pattern of expression suggests an important role of these receptors in modulating the function of mature B cells, as reflected by the increased Ig production following TLR7 stimulation of cells treated with mAb PCT3. Besides, their distribution evidences the existence of further heterogeneity in the populations of effector/memory B cells and plasmablasts defined according to the expression of CD2 and CD21^b^ markers. The study of this heterogeneity may contribute to a better understanding of memory B cell development in swine and provide clues to improve vaccination strategies.
